# Body modification in university students: Attitudes and role of personal body alteration experience

**DOI:** 10.1192/j.eurpsy.2021.1991

**Published:** 2021-08-13

**Authors:** E. Vasilieva, E. Nikolaev, D. Mengeliyeva, E. Nikolaev, S. Petunova, Y. Petunova

**Affiliations:** 1 Social And Clinical Psychology, Ulianov Chuvash State University, Cheboksary, Russian Federation; 2 Medical Faculty, Ulianov Chuvash State University, Cheboksary, Russian Federation; 3 Social And Clinical Psychology, Ulianov Chuvash State University, Chebokasry, Russian Federation; 4 General Medicine Faculty, I.M. Sechenov First Moscow State Medical University (Sechenov University), Moscow, Russian Federation

**Keywords:** Body modifications, response to stress, University Students, mental status

## Abstract

**Introduction:**

Body modifications are a common practice in altering one’s appearance. Some authors refer to such practices body injuring (tattooing, piercing) and indirect body modification (dieting, bodybuilding).

**Objectives:**

To study the attitudes of university students to body modifications considering their personal adaptation potential and experience of body injuring when modifying it.

**Methods:**

We surveyed 104 university students aged 17–24 (65.3% males). The first group included 52 students who had experienced body altering (tattooing, piercing), the second group – 52 students without such an experience. We used the Maddi Hardiness Scale to assess the personal adaptation potential and a 14-point questionnaire to estimate the attitude to body modification.

**Results:**

Over the half of the students in both groups consider that an insufficiently beautiful body needs “improving” (63.4% и 51.9%), but people do not have to intensively build up their muscles (51.9% и 84.7%). Students with modified bodies look more positively at piercing (z=5.4; p=.0001), weight control (z=5.20; p=.0001) and plastic surgery (z=4.02; p=.0001). Students with unmodified bodies credibly more rarely regard tattoo as decoration (z=3.7; p=.0002) and have a more negative attitude to pediatricians having tattoos (z=2.9; p=.003). Indicators of psychological hardiness in the first group are credibly lower – commitment (р=.01), control (р=.001) and challenge (р=.0001).

**Conclusions:**

Students with a higher adaptation potential limit themselves to indirect body modifications (physical exercises). Students with a lower adaptation potential more often resort to body injuring (tattooing, piercing), which may reflect peculiarities of their personal response to stress or peculiarities of their mental status.

**Disclosure:**

No significant relationships.

